# Supplementary material for: A 4-Hydroxybenzoic Acid-Mediated Signaling System Controls the Physiology and Virulence of Shigella sonnei
Source: Microbiol Spectr. 2023 Apr 10;11(3):e04835-22. doi: 10.1128/spectrum.04835-22 (PMC10269604; doi:10.1128/spectrum.04835-22)
Supplement: Supplemental file 1 — Fig. S1 to S13 and Tables S1 to S6. Download spectrum.04835-22-s0001.pdf, PDF file, 1.7 MB [file spectrum.04835-22-s0001.pdf]

Supplementary Information

A 4-hydroxybenzoic acid-mediated signaling system controls the physiology and virulence of *Shigella sonnei*

Mingfang Wang[1], Jia Zeng[1], Yu Zhu[1], Xiayu Chen[1], Quan Guo[1], Huihui Tan[1], Binbin Cui[1], Shihao Song[1,2], Yinyue Deng[1,2]*

[1]*School of Pharmaceutical Sciences (Shenzhen), Shenzhen Campus of Sun Yat-sen University, Sun Yat-sen University, Shenzhen 518107, China*

[2] *School of Pharmaceutical Sciences, Hainan University, Haikou 570228, China*

*To whom correspondence may be addressed.

Email: Yinyue Deng: dengyle@mail.sysu.edu.cn

Running title: 4-HBA controls *Shigella sonnei* physiology and virulence

This file includes:

Supplementary Figures S1 to S13

Supplementary Tables S1 to S6

SUPPLEMENTARY FIGURES AND TABLES

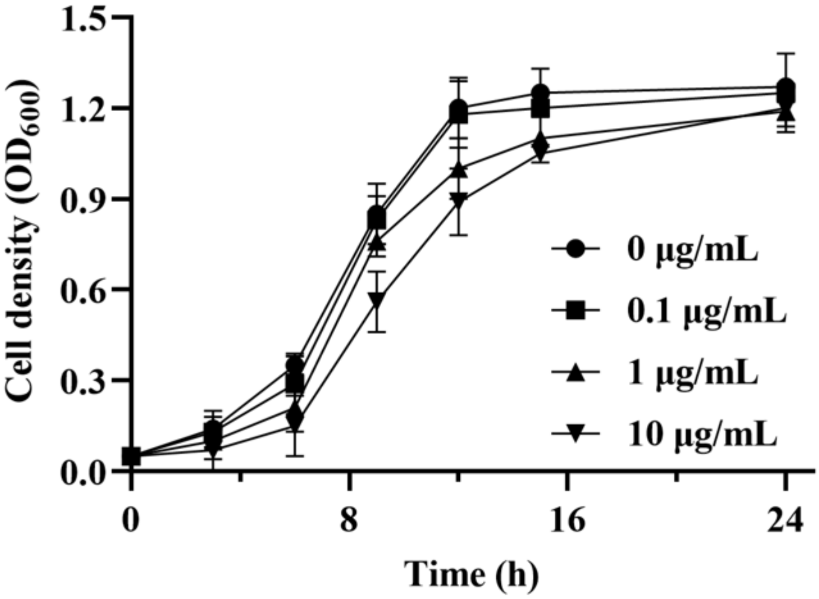

Fig. S1 The growth curve of *C. albicans* in the absence and presence of the extract of *S. sonnei*. The data are presented as the means ± SD of three independent experiments. Error bars indicate SDs.

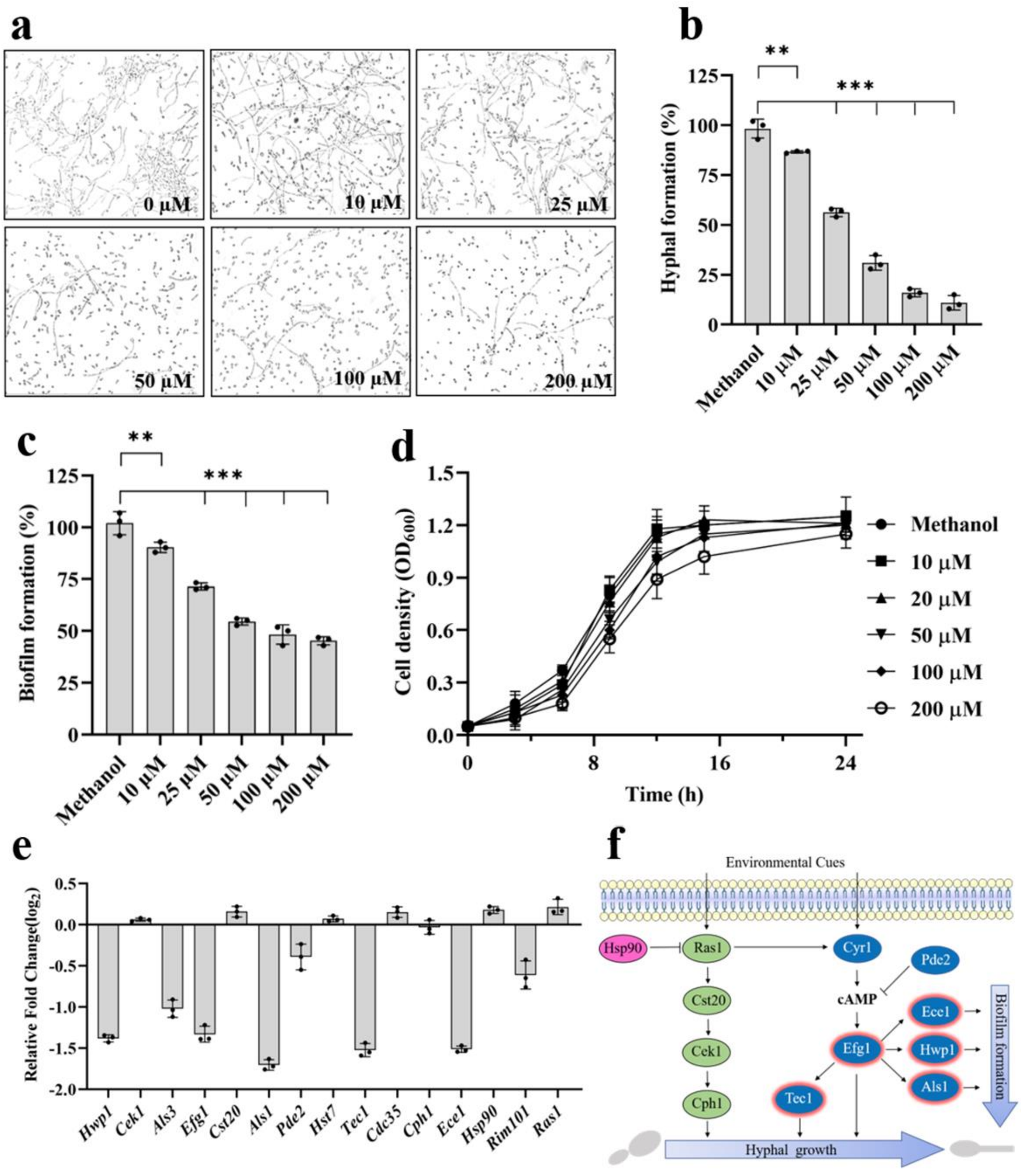

Fig. S2 Influences of 4-HBA on *C. albicans*. Effects of 4-HBA on hyphal growth (a, b) and biofilm formation (c) of *C. albicans*. For convenient comparison, the value of the wild-type strain was defined as 100% to normalize the ratios of the samples treated with different concentrations of 4-HBA. (d) The growth curve of *C. albicans* treated with 0, 10, 25, 50, 100, and 200 μM 4-HBA. (e) The RT–qPCR results showed that the expression levels of genes related to hyphal formation and biofilm formation that were treated with 50 μM 4-HBA were significantly downregulated. (f) Schematic diagram of the signal transduction pathways involved in the mitogen-activated protein kinase (MAPK) pathway (green) and the cyclic AMP-dependent pathway (blue). The red border indicates that the expression level of the gene encoding this protein was significantly inhibited by 4-HBA. The data are presented as the means ± SD of three independent experiments. Error bars indicate the SDs. The significance

of the results was determined by one-way ANOVA (*p <0.05; **p < 0.01; ***p < 0.001; ns = no significance).

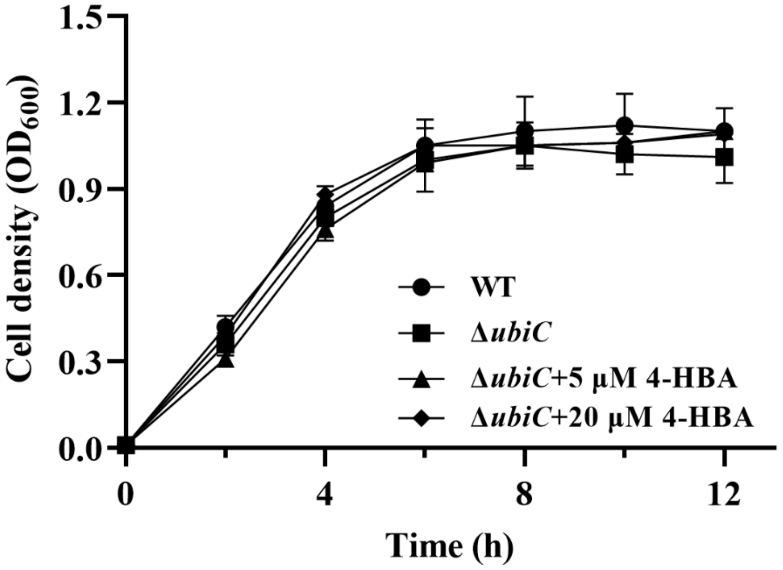

Fig. S3 The growth curve of *S. sonnei*, the *ubiC* mutant, and the *ubiC* mutant with the addition of different concentrations of 4-HBA. The data are presented as the means ± SD of three independent experiments. Error bars indicate the SDs.

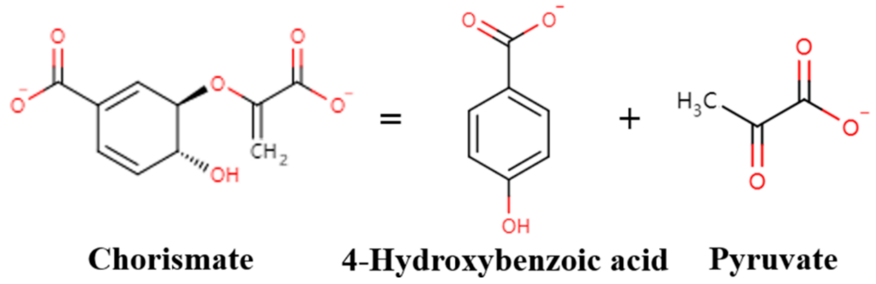

Fig. S4 Catalytic reaction of UbiC.

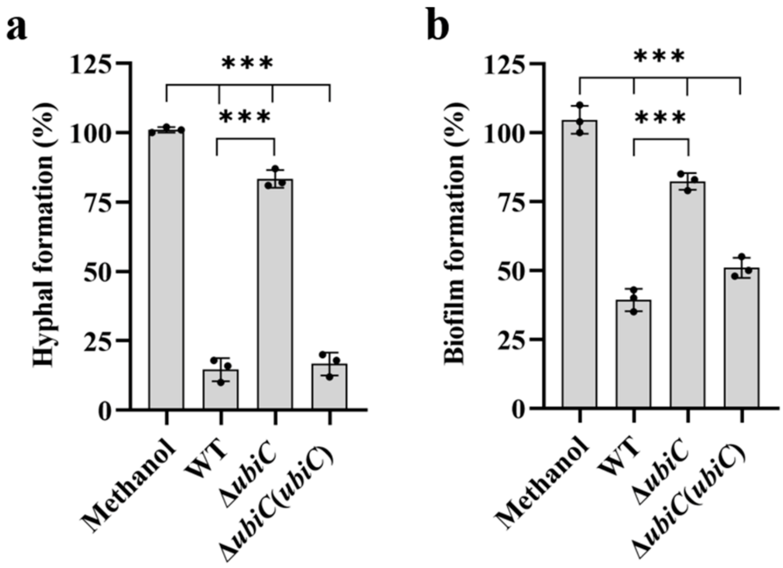

Fig. S5 Effects of the extract of *S. sonnei* on *C. albicans*. The effect on hyphal growth (a) and biofilm formation (b) of *C. albicans* treated with the extract of *S. sonnei* wild-type, *ubiC* deletion mutant, and complemented strains. The final concentration of the extract was 10 μg/mL, which was dissolved in methanol, and the same volume of methanol (used as the solvent for the extract) was used as a control. For convenient comparison, the value of *C. albicans* treated with methanol was defined as 100% to normalize the ratios of the samples treated with the extracts. The data are presented as the means ± SD of three independent experiments. Error bars indicate the SDs. The significance of the results was determined by one-way ANOVA (**p* <0.05; ***p* < 0.01; ****p* < 0.001; ns = no significance).

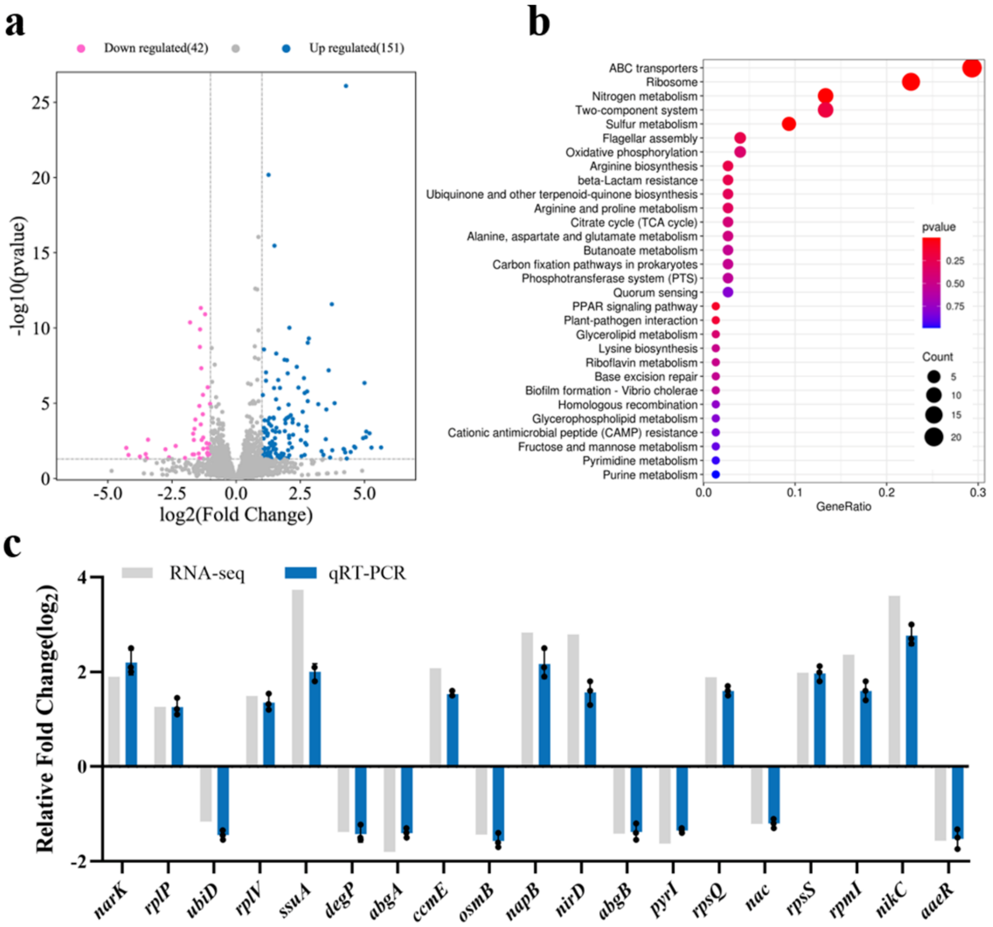

Fig. S6 Differential gene expression profiles between the *ubiC* mutant strain and the wild-type strain as measured by RNA-Seq (Log_2 fold change ≥1). (a) The number of genes upregulated and downregulated in the Δ*ubiC* strain compared with the wild-type strain. (b) GeneRatio analysis of differentially expressed genes between the Δ*ubiC* strain and the wild-type strain. (c) RT–qPCR analysis of the genes that showed differential expression in the Δ*ubiC* strain compared with the wild-type strain. The data are presented as the means ± SD of three independent experiments. Error bars indicate the SDs.

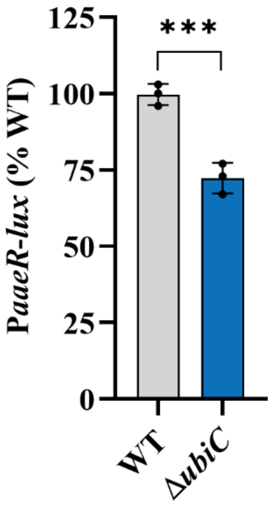

Fig. S7 Analysis of transcriptional expression of *aaeR* in the *ubiC* deletion mutant strain. The gene expression levels of *aaeR* were evaluated by assessing the light production of the *aaeR-luxCDABE* transcriptional fusions in the *S. sonnei* strains. The value of the wild-type strain was defined as 100% to normalize the ratios of the *ubiC* deletion mutant strain. The data are presented as the means ± SD of three independent experiments. Error bars indicate the SDs. The significance of the results was determined by one-way ANOVA (**p* <0.05; ***p* < 0.01; ****p* < 0.001; ns = no significance).

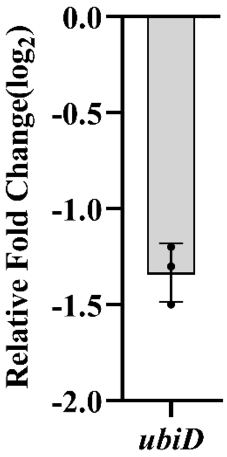

Fig. S8 RT–qPCR analysis of transcriptional expression of *ubiD* in the Δ*aaeR* strain compared with that in the wild-type strain. The data are presented as the means ± SD of three independent experiments. Error bars indicate the SDs.

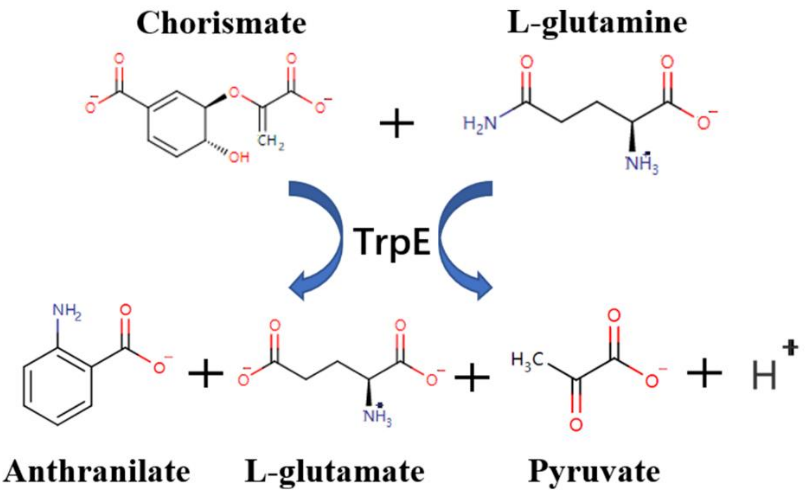

Fig. S9 Catalytic reaction of TrpE.

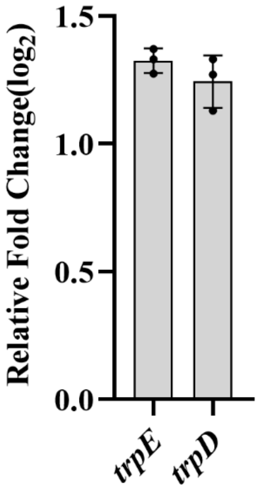

Fig. S10 RT–qPCR analysis of transcriptional expression of *trpE* and *trpD* in the Δ*ubiC* strain compared with that in the wild-type strain. The data are presented as the means ± SD of three independent experiments. Error bars indicate the SDs.

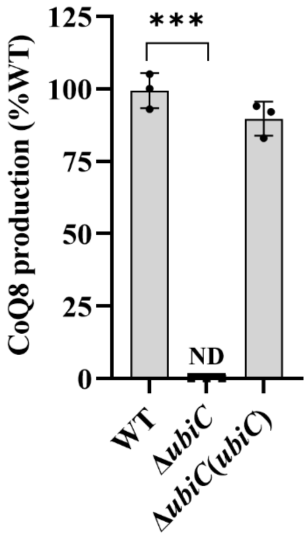

Fig. S11 Detection of CoQ8 production via the LC–MS assay. For convenient comparison, CoQ8 production in the *S. sonnei* wild-type strain was arbitrarily defined as 100% and used to normalize the production ratios of other strains. The data are presented as the means ± SD of three independent experiments. Error bars indicate the SDs. The significance of the results was determined by one-way ANOVA (**p* <0.05; ***p* < 0.01; ****p* < 0.001; ns = no significance). ND, not detected.

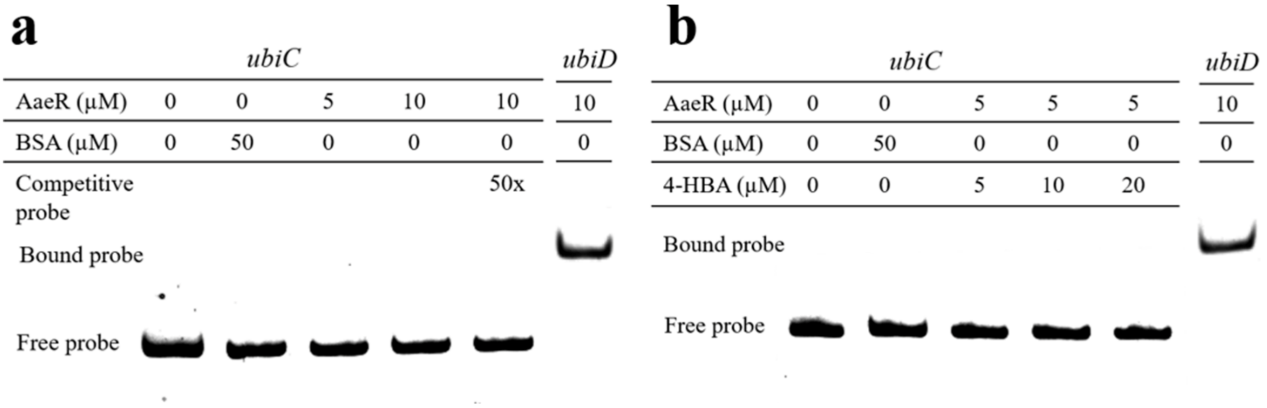

Fig. S12 Analysis of the interaction between AaeR and *ubiC* promoters. (a) EMSA analysis of the binding of AaeR to the *ubiC* promoters. (b) The effects of 4-HBA on the binding of AaeR to the promoters of *ubiC.* The EMSA analysis of the binding of AaeR to the *ubiD* promoters was used as a positive control. A protein–DNA complex, represented by a band shift, was formed when the AaeR protein was incubated with the probe at room temperature for 30 min.

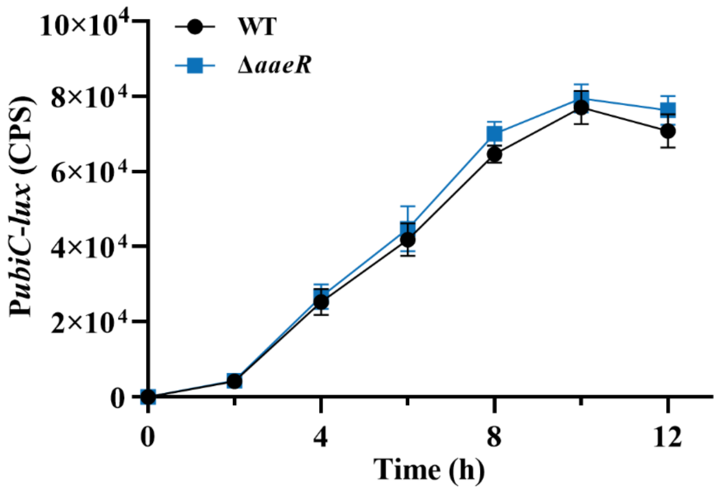

Fig. S13 The gene expression levels of *ubiC* in the *S. sonnei* wild-type strain and the *aaeR* deletion mutant strain. The gene expression levels of *ubiC* were evaluated by assessing the light production of the *ubiC-luxCDABE* transcriptional fusions in the *S. sonnei* strains. The data are presented as the means ± SD of three independent experiments. Error bars indicate the SDs.

Supplementary Table 1 ^{1}H NMR (400 MHz) and ^{13}C NMR (101 MHz) data of 4-Hydroxybenzoic acid in CD_3OD.

NO.	4-Hydroxybenzoic acid	
	δ_C	δ_H
1	122.7	
2/6	133.0	7.83
3/5	116.0	6.77
4	163.4	
7	170.1	

Supplementary Table 2 List of genes differentially expressed in the *ubiC* mutant compared to the wild-type strain (Log_2 fold change ≥ 1). Significantly differentially expressed genes were determined by Cufflinks after Benjamini-Hochberg correction. The fold change is the ratio of the mutant FPKM to the wild-type FPKM

Gene id	Name	Log_2FC	Description
COO90_09400	*ssuE*	4.28	NADPH-dependent FMN reductase
COO90_22945	*rplP*	1.26	50S ribosomal protein L16
COO90_22955	*rplV*	1.49	50S ribosomal protein L22
COO90_09395	*ssuA*	3.73	aliphatic sulfonate ABC transporter substrate-binding protein SsuA
COO90_05545	*degP*	-1.38	serine endoprotease DegP
COO90_15505	*nac*	-1.21	nitrogen assimilation transcriptional regulator
COO90_14080	*abgA*	-1.80	p-aminobenzoyl-glutamate hydrolase subunit AbgA
COO90_16595	*ccmE*	2.08	cytochrome c maturation protein CcmE
COO90_22720		-1.41	amino acid ABC transporter substrate-binding protein
COO90_16625	*napB*	2.83	nitrate reductase cytochrome c-type subunit
COO90_23170	*nirD*	2.79	nitrite reductase small subunit NirD
COO90_14085	*abgB*	-1.41	p-aminobenzoyl-glutamate hydrolase subunit AbgB
COO90_02275	*glpK*	1.08	glycerol kinase GlpK
COO90_16585	*dsbE*	1.56	thiol:disulfide interchange protein DsbE
COO90_22935	*rpsQ*	1.89	30S ribosomal protein S17
COO90_22960	*rpsS*	1.98	30S ribosomal protein S19
COO90_12125	*rpmI*	2.36	50S ribosomal protein L35
COO90_17855		-1.36	DUF2502 domain-containing protein
COO90_23780	*nikC*	3.61	nickel ABC transporter permease subunit NikC
COO90_01860	*ubiD*	-1.16	4-hydroxy-3-polyprenylbenzoate decarboxylase
COO90_23185		2.01	YhfL family protein
COO90_16640	*napA*	2.64	periplasmic nitrate reductase subunit alpha

COO90_16605	*ccmC*	1.64	heme exporter protein CcmC
COO90_02925	*ubiA*	1.16	4-hydroxybenzoate octaprenyltransferase
COO90_06380	*tauA*	5.00	taurine ABC transporter substrate-binding protein
COO90_03170	*yjdP*	-1.11	protein YjdP
COO90_16615	*ccmA*	2.43	cytochrome c biogenesis heme-transporting ATPase CcmA
COO90_22970	*rplW*	1.71	50S ribosomal protein L23
COO90_03790	*priB*	1.48	primosomal replication protein N
COO90_23775	*nikD*	2.77	nickel import ATP-binding protein NikD
COO90_16630	*napH*	2.67	quinol dehydrogenase ferredoxin subunit NapH
COO90_08830		-1.29	CHASE9 sensor domain-containing protein
COO90_17965	*ptsH*	1.04	phosphocarrier protein Hpr
COO90_08710		1.56	hypothetical protein
COO90_16635	*napG*	2.79	ferredoxin-type protein NapG
COO90_06385	*tauB*	3.84	taurine ABC transporter ATP-binding subunit
COO90_06430		-1.02	DUF2755 family protein
COO90_23785	*nikB*	3.20	nickel ABC transporter permease subunit NikB
COO90_16620	*napC*	2.04	cytochrome c-type protein NapC
COO90_14420	*osmB*	-1.43	osmotically-inducible lipoprotein OsmB
COO90_02735	*aceB*	1.66	malate synthase A
COO90_10115		3.52	IS3-like element IS2 family transposase
COO90_15000	*narJ*	2.59	nitrate reductase molybdenum cofactor assembly chaperone
COO90_08205		-1.30	hypothetical protein
COO90_14995	*narI*	2.13	respiratory nitrate reductase subunit gamma
COO90_23765	*nikR*	2.05	nickel-Responsive transcriptional regulator NikR
COO90_16590	*ccmF*	1.46	cytochrome c-type biogenesis heme lyase CcmF
COO90_15010		2.00	nitrate reductase subunit alpha
COO90_10865		1.30	DUF2766 domain-containing protein
COO90_03795	*rpsR*	1.97	30S ribosomal protein S18
COO90_20980		1.47	energy-coupling factor ABC transporter ATP-binding protein
COO90_08035	*sdhD*	2.21	succinate dehydrogenase membrane anchor subunit
COO90_16370		-1.59	LysR family transcriptional regulator
COO90_16610	*ccmB*	1.91	heme exporter protein CcmB
COO90_22915	*rpsN*	1.11	30S ribosomal protein S14
COO90_12575	*nth*	1.24	endonuclease III
COO90_15005	*narH*	2.20	nitrate reductase subunit beta

COO90_23165	*nirB*	1.93	NADPH-nitrite reductase large subunit
COO90_00660	*lldP*	1.29	L-lactate permease
COO90_07700	*gltL*	-1.38	glutamate/aspartate ABC transporter ATP binding protein GltL
COO90_23175	*nirC*	2.43	nitrite transporter NirC
COO90_09925		1.37	FTR1 family protein
COO90_22890	*rpmD*	2.74	50S ribosomal protein L30
COO90_02230	*sbp*	1.57	sulfate/thiosulfate ABC transporter substrate-binding protein Sbp
COO90_12585	*rsxG*	1.08	electron transport complex subunit RsxG
COO90_04000	*pyrI*	-1.62	aspartate carbamoyltransferase regulatory subunit
COO90_13580	*hicB*	1.17	type II toxin-antitoxin system antitoxin HicB
COO90_07925		1.42	hypothetical protein
COO90_23770	*nikE*	2.75	nickel import ATP-binding protein NikE
COO90_15015	*narK*	1.90	nitrate transporter NarK
COO90_24095	*yidD*	5.07	membrane protein insertion efficiency factor YidD
COO90_20865	*ssrS*	1.53	6S RNA
COO90_17035	*nuoK*	1.69	NADH-quinone oxidoreductase subunit NuoK
COO90_20220	*mazF*	1.06	endoribonuclease MazF
COO90_22900	*rplR*	1.13	50S ribosomal protein L18
COO90_14130		5.20	IS1-like element IS1A family transposase
COO90_20510	*lysA*	1.23	diaminopimelate decarboxylase
COO90_15135		-1.67	IS1-like element IS1A family transposase
COO90_19140		1.22	phage tail assembly protein
COO90_07095	*ybcJ*	2.22	ribosome-associated protein YbcJ
COO90_06860	*ffs*	2.06	signal recognition particle sRNA small type
COO90_04505	*rimI*	1.21	ribosomal protein S18-alanine N-acetyltransferase
COO90_22075	*prlF*	5.06	type II toxin-antitoxin system antitoxin PrlF
COO90_14925	*oppB*	-1.28	oligopeptide ABC transporter permease OppB
COO90_03110	*nrfF*	2.07	heme lyase NrfEFG subunit NrfF
COO90_23395	*feoC*	1.51	[Fe-S]-dependent transcriptional repressor FeoC
COO90_05965		4.95	ISAs1 family transposase
COO90_22925	*rplX*	1.07	50S ribosomal protein L24
COO90_19895	*hypC*	3.50	hydrogenase 3 maturation protein HypC
COO90_02010		-3.44	IS4-like element IS4 family transposase
COO90_01950		-1.65	IS1 family transposase
COO90_17015		-1.34	YfbM family protein
COO90_09390	*ssuD*	2.52	FMNH2-dependent alkanesulfonate

			monooxygenase
COO90_15590		2.69	BMC domain-containing protein
COO90_04075		1.31	IS1-like element IS1A family transposase
COO90_01990	*glnG*	-1.15	nitrogen regulation protein NR(I)
COO90_08865		2.09	IS3-like element IS600 family transposase
COO90_21090		-1.05	type IV pilus twitching motility protein PilT
COO90_16650	*napF*	1.46	ferredoxin-type protein NapF
COO90_22600	*AaeR*	-1.52	LysR family transcriptional regulator AaeR
COO90_06790		2.74	IS3 family transposase
COO90_12580	*rsxE*	1.38	electron transport complex subunit RsxE
COO90_24100	*rnpA*	1.37	ribonuclease P protein component
COO90_14335	*puuA*	1.42	glutamate-putrescine ligase
COO90_07575		-2.36	IS4-like element IS4 family transposase
COO90_24105	*rpmH*	1.44	50S ribosomal protein L34
COO90_21505		4.60	molecular chaperone
COO90_16645	*napD*	5.65	chaperone NapD
COO90_14885		2.18	IS3-like element IS600 family transposase
COO90_14260		5.28	carbohydrate ABC transporter permease
COO90_18825		4.64	IS1-like element IS1A family transposase
COO90_03090	*nrfB*	1.06	cytochrome c nitrite reductase pentaheme subunit
COO90_17565		-4.28	IS3-like element IS2 family transposase
COO90_03095	*nrfC*	1.11	cytochrome c nitrite reductase Fe-S protein
COO90_21630		1.27	IS4-like element IS4 family transposase
COO90_14930	*oppA*	-1.12	oligopeptide ABC transporter substrate-binding protein OppA
COO90_05990	*ivy*	-1.05	C-lysozyme inhibitor
COO90_22680	*fis*	1.47	DNA-binding transcriptional regulator Fis
COO90_14410		1.22	hypothetical protein
COO90_11860	*gdhA*	-1.28	NADP-specific glutamate dehydrogenase
COO90_00320		4.19	IS3-like element IS600 family transposase
COO90_18335		1.55	formate/nitrite transporter family protein
COO90_00705		-2.76	ATP-binding protein
COO90_16600	*ccmD*	4.21	heme exporter protein CcmD
COO90_02280	*glpF*	1.45	glycerol uptake facilitator protein GlpF
COO90_18500	*iscX*	1.09	Fe-S cluster assembly protein IscX
COO90_03230	*phnE*	2.03	phosphonate ABC transporter, permease protein PhnE
COO90_22875	*rpmJ*	3.75	50S ribosomal protein L36
COO90_06390	*tauC*	3.33	taurine ABC transporter permease TauC
COO90_00165	*uhpA*	1.03	transcriptional regulator UhpA
COO90_19325	*rpsP*	1.02	30S ribosomal protein S16
COO90_06130		4.46	IS3-like element IS600 family transposase
COO90_03985	*mgtL*	1.33	*mgtA* regulatory leader peptide MgtL

COO90_08030	*sdhC*	1.24	succinate dehydrogenase cytochrome b556 subunit
COO90_08105	*ybgE*	1.04	*cyd* operon protein YbgE
COO90_12100	*espL1*	4.25	type III secretion system effector EspL1
COO90_07555	*cspE*	1.09	transcription antiterminator/RNA stability regulator CspE
COO90_10740		1.34	GlsB/YeaQ/YmgE family stress response membrane protein
COO90_14090		-1.12	AbgT family transporter
COO90_08915		-1.13	hypothetical protein
COO90_04755		1.54	transposase
COO90_10770		1.77	IS630-like element IS630 family transposase
COO90_00885	*dppB*	-1.03	dipeptide ABC transporter permease DppB
COO90_20555		-1.46	transcriptional regulator
COO90_00505	*rpmG*	1.85	50S ribosomal protein L33
COO90_17505		-3.53	phage tail protein
COO90_00495	*radC*	1.22	DNA repair protein RadC
COO90_10680		1.70	IS110 family transposase
COO90_17750	*yfdV*	-1.64	transporter YfdV
COO90_10245	*flgF*	1.27	flagellar basal-body rod protein FlgF
COO90_13650		3.40	IS1-like element IS1A family transposase
COO90_03155		3.40	hypothetical protein
COO90_17515		-4.20	DNA-packaging protein FI
COO90_08665		2.67	TonB-dependent receptor plug domain-containing protein
COO90_08825	*gsiD*	-1.10	glutathione ABC transporter permease GsiD
COO90_15175	*yedF*	2.07	sulfurtransferase-like selenium metabolism protein YedF
COO90_02085		1.63	IS1-like element IS1A family transposase
COO90_10240	*flgE*	1.24	flagellar hook protein FlgE
COO90_14535	*trpE*	1.39	anthranilate synthase component I
COO90_14540	*trpD*	1.25	anthranilate synthase component II
COO90_22330	*rpmA*	1.04	50S ribosomal protein L27
COO90_08025		1.78	hypothetical protein
COO90_18015	*cysP*	1.64	thiosulfate/sulfate ABC transporter substrate-binding protein CysP
COO90_17920		1.33	RtT sRNA
COO90_02795		2.55	PTS sorbose transporter subunit IIA
COO90_22940	*rpmC*	1.62	50S ribosomal protein L29
COO90_06020	*dinJ*	1.16	type II toxin-antitoxin system antitoxin DinJ
COO90_18620		1.06	sugar ABC transporter ATP-binding protein
COO90_03105		1.08	heme lyase CcmF/NrfE family subunit
COO90_18550		1.32	alpha/beta hydrolase
COO90_14330	puuD	1.78	gamma-glutamyl-gamma-aminobutyrate

			hydrolase
COO90_13440		-2.61	RHS repeat protein
COO90_24055	glpD	1.27	glycerol-3-phosphate dehydrogenase
COO90_07310		1.16	DUF1158 domain-containing protein
COO90_24045		3.66	hypothetical protein
COO90_08440		3.66	IS1 family transposase
COO90_07185	sfmF	1.24	fimbria assembly protein
COO90_15060		1.74	type I toxin-antitoxin system toxin Ldr family protein
COO90_01690		1.85	IS4-like element IS4 family transposase
COO90_09600	hyaC	-2.00	Ni/Fe-hydrogenase b-type cytochrome subunit
COO90_15615	pduP	-1.42	CoA-acylating propionaldehyde dehydrogenase PduP
COO90_13145	safA	1.93	two-component system connector SafA
COO90_04780		1.71	IS1-like element IS1A family transposase
COO90_20570		-3.48	hypothetical protein
COO90_10020	csgA	1.37	curlin major subunit CsgA
COO90_08325		2.02	IS3-like element IS600 family transposase
COO90_07490	citG	2.00	triphosphoribosyl-dephospho-CoA synthase CitG
COO90_06230		-1.30	carbamate kinase family protein
COO90_07495	citX	4.31	citrate lyase holo-[acyl-carrier protein] synthase
COO90_22660		1.84	YhdT family protein
COO90_17485		1.95	phage tail assembly protein T
COO90_10255	flgH	1.84	flagellar basal body L-Ring protein FlgH
COO90_06980		1.86	IS3-like element IS600 family transposase

Supplementary Table 3 Analysis of the homologs of UbiC in various bacteria

Bacteria	Identity (%)	Accession No.
Acinetobacter		
A. apis	33.33	WP_088822924.1
A. baumannii	98.36	WP_147528468.1
A. baylyi	35.29	WP_004928440.1
A. bohemicus	29.33	WP_202737711.1
A. brisouii	27.96	WP_045793966.1
A. calcoaceticus	26.51	WP_199964570.1
A. johnsonii	38.30	WP_005400482.1
A. nosocomialis	98.84	PVA00898.1
A. populi	29.41	MCH4248029.1
A. seifertii	26.51	WP_200027268.1
A. shaoyimingii	30.12	WP_166010869.1
A. silvestris	26.51	WP_086203486.1
A. soli	33.33	WP_004938973.1
A. terrae	28.57	WP_171542548.1
A. terrestris	28.21	WP_131267885.1
Aliivibrio		
A. finisterrensis	31.62	WP_151655644.1
A. fischeri	33.82	WP_155654068.1
A. salmonicida	26.73	WP_012551314.1
A. sifiae	33.09	WP_105064409.1
A. wodanis	32.35	CED72580.1
Azotobacter		
A. beijerinckii	27.45	SEI75117.1
A. chroococcum	27.45	WP_039801292.1
A. salinestris	27.45	WP_152388219.1
A. vinelandii	28.42	WP_061288412.1
Buttiauxella		
B. agrestis	70.37	WP_034492397.1
B. brennerae	68.52	WP_064561703.1
B. gaviniae	69.75	WP_064515363.1
B. izardii	69.33	WP_120064953.1
B. massiliensis	69.75	WP_151994228.1
B. noackiae	69.14	WP_064556042.1
B. warmboldiae	69.75	WP_124024548.1
Cedecea		
C. colo	73.46	WP_167614369.1
C. davisae	72.22	WP_202307063.1
C. lapagei	85.45	PKA29687.1
C. neteri	73.46	WP_039299465.1

C. neteri	72.84	WP_061276041.1
Citrobacter		
C. amalonaticus	89.09	EGT3575156.1
C. braakii	90.30	WP_218710038.1
C. cronae	89.09	MCL5519402.1
C. koseri	88.48	WP_012134646.1
C. portucalensis	89.70	WP_174770814.1
C. rodentium	88.48	WP_012907701.1
C. sedlakii	87.88	WP_211934959.1
C. telavivensis	87.88	WP_152401421.1
C. werkmanii	90.30	EGT0668723.1
C. youngae	90.30	WP_006688307.1
Edwardsiella		
E. anguillarum	49.62	RFT04733.1
E. hoshinae	43.27	WP_070245469.1
E. ictaluri	51.13	WP_015869674.1
E. piscicida	49.62	WP_078057710.1
E. tarda	45.54	WP_035597793.1
Enterobacter		
E. asburiae	89.09	WP_196351300.1
E. bugandensis	87.88	WP_248113486.1
E. cancerogenus	85.45	WP_137273377.1
E. chengduensis	86.67	WP_248103900.1
E. cloacae	87.27	WP_046887571.1
E. hormaechei	98.18	QLW04779.1
E. timonensis	83.03	WP_061706855.1
E. wuhouensis	89.09	WP_131635380.1
Franconibacter		
F. helveticus	74.55	WP_024553577.1
F. pulveris	73.94	WP_029593165.1
Pseudomonas		
P. aeruginosa	78.41	MBN0734143.1
P. alcaligenes	34.31	MBB4818033.1
P. allii	35.29	WP_205347593.1
P. anguilliseptica	33.09	WP_233683206.1
P. azadiae	33.00	MBV4453461.1
P. benzenivorans	31.37	WP_090447320.1
P. borbori	32.69	WP_090499825.1
P. canavaninivorans	33.00	WP_258648540.1

P. cavernae	33.33	WP_119895516.1
P. cavernicola	34.31	WP_119953179.1
P. cedrina	33.00	WP_076952074.1
P. chlororaphis	34.00	WP_047739420.1
P. citronellolis	33.00	WP_082936943.1
P. corrugata	34.00	WP_024777876.1
P. daroniae	28.43	WP_131179968.1
P. delhiensis	32.00	SDK78578.1
P. dryadis	30.00	WP_131175581.1
P. fildesensis	33.00	WP_048723542.1
P. flexibilis	38.89	WP_234702700.1
P. fluorescens	35.29	WP_058424279.1
P. fulva	34.31	WP_013789404.1
P. haemolytica	33.62	WP_153837568.1
P. leptonychotis	31.37	WP_136663633.1
P. lurida	33.00	WP_222943470.1
P. marginalis	34.00	WP_253400156.1
P. nanhaiensis	31.37	WP_223653613.1
P. oligotrophica	32.35	WP_237256567.1
P. otitidis	35.29	WP_243899425.1
P. peli	32.35	WP_090250763.1
P. pohangensis	32.33	WP_090199180.1
P. punonensis	31.37	WP_073263106.1
P. reactans	56.20	NWA39673.1
P. rhodesiae	35.29	WP_221433540.1
P. salmasensis	33.00	WP_186607089.1
P. schmalbachii	31.37	WP_236032914.1
P. segetis	38.27	WP_089360367.1
P. seleniipraecipitans	35.29	WP_092367305.1
P. simiae	33.00	WP_222942608.1
P. songnenensis	33.33	WP_122098807.1
P. straminea	32.35	WP_093500580.1
P. synxantha	34.31	WP_057023303.1
P. tohonis	34.31	WP_173174862.1
P. trivialis	35.34	WP_049710900.1
P. uvaldensis	34.00	WP_232776951.1
P. viridiflava	32.35	WP_122539207.1
P. yamanorum	35.29	WP_177112353.1
Klebsiella		
K. aerogenes	78.06	SFX92607.1
K. africana	79.39	WP_136029726.1
K. grimontii	81.82	WP_224236096.1
K. huaxiensis	81.21	WP_112215900.1

K. michiganensis	81.21	WP_160743330.1
K. oxytoca	98.79	SBL11794.1
K. pasteurii	81.21	WP_142470369.1
K. pneumoniae	99.39	MBA0013941.1
K. quasipneumoniae	81.82	WP_117125007.1
K. quasivariicola	78.79	WP_224423626.1
K. spallanzanii	82.42	WP_139540812.1
K. variicola	79.39	WP_110198128.1
Kluyvera		
Kluyvera ascorbata	78.18	HAT3955248.1
Kluyvera cryocrescens	75.76	WP_061283371.1
Kluyvera cryocrescens	66.67	VFS69057.1
Kluyvera genomosp. 1	73.94	WP_052281824.1
Kluyvera georgiana	80.61	WP_065357785.1
Kluyvera intermedia	83.03	WP_085005890.1
Kluyvera sichuanensis	78.79	WP_185668446.1
Kosakonia		
K. arachidis	80.61	WP_090121807.1
K. cowanii	84.24	WP_139966823.1
K. oryzae	81.21	WP_064568814.1
K. oryzendophytica	84.85	WP_061498857.1
K. oryziphila	81.82	WP_090136788.1
K. pseudosacchari	86.67	WP_086873908.1
K. quasisacchari	86.67	WP_131407001.1
K. radicincitans	82.42	WP_043955711.1
Leclercia		
L. adecarboxylata	88.48	KML21150.1
L. sp. LSNIH3	81.82	AUY39165.1
Lelliottia		
L. amnigena	86.06	WP_202665646.1
L. nimipressuralis	88.48	TFB29656.1
L. sp. WAP21	85.45	WP_230352986.1
Mangrovibacter		
M. phragmitis	68.07	WP_064596768.1
M. plantisponsor	68.07	WP_110026587.1
M. sp. MFB070	68.07	WP_036102985.1
M. yixingensis	68.07	WP_226574178.1
Photobacterium		

P. alginatilyticum	36.76	WP_160652482.1
P. andalusiense	33.82	WP_087854332.1
P. angustum	33.82	WP_105061175.1
P. aphoticum	38.97	GHA43539.1
P. aquae	33.98	WP_047877737.1
P. aquimaris	33.82	WP_060997736.1
P. atrarenae	36.76	WP_255389100.1
P. carnosum	31.00	WP_232601400.1
P. chitinilyticum	31.00	WP_128785038.1
P. damselae	38.97	WP_065172477.1
P. frigidiphilum	31.00	WP_107241993.1
P. gaetbulicola	35.29	WP_044622288.1
P. galatheae	41.38	WP_081819569.1
P. ganghwense	31.00	WP_217392417.1
P. halotolerans	45.16	WP_161444863.1
P. iliopiscarium	30.00	WP_045035868.1
P. indicum	32.00	WP_107254485.1
P. jeanii	36.76	WP_068330465.1
P. kishitanii	33.09	CEO37822.1
P. leiognathi	37.50	WP_107166735.1
P. lipolyticum	29.00	WP_107283546.1
P. lutimaris	33.00	WP_107349726.1
P. malacitanum	35.29	WP_087845740.1
P. marinum	38.24	WP_007469524.1
P. phosphoreum	34.00	WP_232605387.1
P. piscicola	34.56	SKC34354.1
P. profundum	32.00	WP_006233275.1
P. proteolyticum	36.03	WP_075766277.1
P. rosenbergii	31.00	WP_107298931.1
P. salinisoli	45.16	WP_120513687.1
P. sanctipauli	37.5	WP_036818650.1
P. sanguinicancri	31.00	WP_062688249.1
P. swingsii	31.00	WP_048898785.1
P. toruni	34.56	WP_080174279.1
Phytobacter		
P. palmae	83.64	SFF31193.1
P. ursingii	83.03	VTP16112.1
P. massiliensis	80.00	WP_044180994.1
Raoultella		
R. ornithinolytica	80.00	WP_099842587.1
R. planticola	80.61	WP_143718830.1
R. terrigena	76.53	ROS14953.1

Salinivibrio		
S. siamensis	34.56	WP_077667392.1
S. socompensis	37.50	WP_025673764.1
S. costicola	40.43	WP_167313814.1
S. sharmensis	45.74	WP_077771852.1
Salmonella		
S. enterica	86.67	WP_080225204.1
Vibrio		
V. aestuarianus	33.09	CAH8218117.1
V. agarilyticus	36.30	WP_168836726.1
V. algicola	33.82	WP_153445565.1
V. alginolyticus	32.35	KOE81006.1
V. algivorus	35.46	WP_089125151.1
V. campbellii	34.56	WP_255932995.1
V. caribbeanicus	39.02	WP_009602602.1
V. chagasii	34.56	PQJ55708.1
V. cholerae	31.62	WP_199368280.1
V. coralliilyticus	32.35	WP_038514512.1
V. crassostreae	34.56	TCN92470.1
V. diabolicus	34.56	WP_257888792.1
V. fortis	35.29	WP_191907646.1
V. gangliei	43.62	WP_105903026.1
V. harveyi	34.56	WP_239970315.1
V. hepatarius	35.29	WP_215987777.1
V. litoralis	35.46	WP_244875211.1
V. natriegens	33.09	WP_176293244.1
V. neptunius	32.35	WP_045975676.1
V. nitrifigilis	35.77	WP_196124002.1
V. owensii	33.82	AYO13065.1
V. palustris	33.82	WP_077315494.1
V. parahaemolyticus	89.70	KKF71316.1
V. pectenicida	33.09	WP_171361371.1
V. pelagius	35.29	WP_255229647.1
V. rotiferianus	33.82	NOH69051.1
V. rumoiensis	37.59	WP_231897466.1
V. splendidus	34.56	WP_004740537.1
V. tritonius	34.56	WP_068715615.1
V. vulnificus	56.39	HAS8352407.1
V. xuii	32.35	KOO12986.1
Yersinia		

Y. aldovae	62.12	WP_145561849.1
Y. aleksiciae	62.88	WP_145492397.1
Y. enterocolitica	61.36	WP_050917371.1
Y. frederiksenii	60.40	WP_050296004.1
Y. intermedia	62.88	WP_145549664.1
Y. massiliensis	64.39	WP_145513925.1
Y. mollaretii	62.12	WP_050536989.1
Y. nurmii	53.01	WP_049598056.1
Y. pekkanenii	61.36	WP_049613832.1
Y. pestis	60.61	WP_054104465.1
Y. pseudotuberculosis	60.61	WP_050117587.1
Y. rochesterensis	61.36	WP_145591097.1
Y. rohdei	62.88	WP_032817534.1
Y. ruckeri	60.61	WP_145498326.1
Y. similis	60.61	WP_025382305.1
Y. thracica	62.12	WP_050115852.1

Supplementary Table 4 Analysis of the homologs of AaeR in various bacteria

Bacteria	Identity (%)	Accession No.
Acinetobacter		
A. baumannii	30.34	WP_075874607.1
A. calcoaceticus	34.02	WP_035361027.1
A. guillouiae	33.91	WP_234623584.1
A. haemolyticus	32.76	WP_161417858.1
A. junii	33.10	WP_026057409.1
A. lactucae	30.8	WP_200042749.1
A. marinus	34.84	WP_092619727.1
A. nosocomialis	31.49	WP_151687118.1
A. oleivorans	30.80	WP_144730870.1
A. pittii	31.14	WP_068565387.1
A. proteolyticus	34.25	WP_101236130.1
A. qingfengensis	33.45	WP_070070973.1
A. seifertii	30.80	WP_151761171.1
A. stercoris	32.08	WP_121974905.1
A. wuhouensis	32.63	WP_087551875.1
Aliivibrio		
A. finisterrensis	31.56	WP_130087893.1
A. fischeri	35.54	WP_065623086.1
A. logei	33.10	WP_017022582.1
A. salmonicida	32.52	WP_012549938.1
A. sifiae	33.45	WP_060991991.1
A. wodanis	33.10	CED56831.1
Azotobacter		
A. beijerinckii	37.58	WP_090619540.1
A. chroococcum	37.06	WP_131348511.1
A. salinestris	35.57	WP_152388762.1
A. vinelandii	36.91	WP_012699727.1
Buttiauxella		
B. agrestis	84.26	WP_115627132.1
B. brennerae	84.56	WP_064561038.1
B. ferragutiae	33.80	WP_232887014.1
B. gaviniae	84.59	WP_064514148.1
B. izardii	84.92	WP_120064619.1
B. massiliensis	86.73	WP_151992953.1
B. noackiae	84.90	WP_034459827.1
B. warmboldiae	84.59	WP_124024170.1
Cedecea		

C. colo	86.93	WP_167614548.1
C. davisae	84.64	WP_202306180.1
C. lapagei	90.40	PKA30395.1
C. neteri	85.53	WP_039298229.1
Citrobacter		
C. amalonaticus	95.38	EGT3574168.1
C. braakii	93.40	HCB1811990.1
C. farmeri	94.39	WP_216526323.1
C. freundii	96.04	WP_182270902.1
C. koseri	95.38	HAU5603233.1
C. pasteurii	94.06	CEJ67695.1
C. portucalensis	93.73	WP_149335570.1
C. rodentium	94.06	WP_012908473.1
C. sedlakii	95.05	WP_211935185.1
C. telavivensis	94.06	WP_152406187.1
C. tructae	95.05	WP_135324132.1
C. werkmanii	94.06	EGT0668653.1
C. youngae	94.06	WP_032940709.1
Edwardsiella		
E. anguillarum	25.68	AIJ09208.1
E. hoshinae	24.90	WP_035370342.1
E. ictaluri	67.46	WP_015872690.1
E. piscicida	41.79	WP_226064110.1
E. tarda	67.46	BAU80611.1
Enterobacter		
E. bugandensis	90.73	WP_252016337.1
E. cancerogenus	90.07	HBI6868644.1
E. cloacae	94.06	SAE28802.1
E. hormaechei	91.39	WP_193129820.1
E. huaxiensis	90.07	WP_119936781.1
E. kobei	90.40	WP_121527298.1
E. oligotrophicus	90.07	WP_152082456.1
E. quasimori	90.40	WP_126545354.1
E. timonensis	90.37	WP_061707028.1
E. wuhouensis	90.40	WP_131635267.1
Franconibacter		
F. daqui	31.86	GGD36673.1
F. helveticus	87.38	WP_110877607.1
F. pulveris	86.71	WP_024560075.1
F. sp. IITDAS19	33.91	WP_247365337.1

Pseudomonas		
P. aeruginosa	90.73	MBH4409277.1
P. alcaligenes OT 69	34.28	EQM69117.1
P. alcaliphila	32.75	WP_075745530.1
P. anguilliseptica	35.86	WP_090376614.1
P. boanensis	35.40	WP_215377724.1
P. chengduensis	32.75	WP_064494030.1
P. composti	34.49	WP_036999256.1
P. fluvialis	33.69	WP_184683497.1
P. hydrolytica	33.80	WP_129482875.1
P. indoloxydans	36.84	WP_108234435.1
P. insulae	36.49	WP_205350025.1
P. khazarica	36.18	WP_134676618.1
P. lalkuanensis	35.69	WP_151137319.1
P. mangiferae	36.75	WP_143486663.1
P. mendocina	33.80	WP_148117426.1
P. oleovorans	36.18	WP_037054751.1
P. oligotrophica	35.49	WP_237257269.1
P. reactans	80.27	NWA36542.1
P. resinovorans	34.71	WP_077526331.1
P. taiwanensis	35.40	WP_179059682.1
P. tohonis	34.36	WP_236204284.1
P. typographi	33.22	WP_194718645.1
P. ullengensis	35.46	WP_183086969.1
P. viridiflava	34.28	WP_122450723.1
Klebsiella		
K. aerogenes	90.73	BCZ64383.1
K. africana	89.67	WP_136031022.1
K. huaxiensis	89.00	WP_112213525.1
K. oxytoca	89.67	WP_174509411.1
K. pneumoniae	90.40	SSH37804.1
K. quasipneumoniae	91.06	TYF84814.1
K. quasivariicola	89.67	WP_224423957.1
K. spallanzanii	89.33	WP_139540730.1
K. variicola	90.33	WP_039102584.1
Kluyvera		
K. ascorbata	90.40	EJD5396400.1
K. cryocrescens	89.11	WP_061281577.1
K. genomosp. 1	89.11	WP_052284002.1
K. georgiana	90.40	WP_064541759.1
K. intermedia	90.70	WP_153742058.1

K. sichuanensis	90.40	WP_185669799.1
Kosakonia		
K. arachidis	86.96	WP_090121673.1
K. cowanii	89.63	WP_076769262.1
K. oryzae	89.30	WP_064563346.1
K. oryzendophytica	90.64	WP_061492968.1
K. oryziphila	86.96	WP_090136513.1
K. pseudosacchari	33.91	PDO82804.1
K. quasisacchari	90.30	WP_131407853.1
K. radicincitans	88.63	WP_035887998.1
K. sacchari	32.20	SEK86323.1
Leclercia		
L. adecarboxylata	90.07	KML23469.1
L. pneumoniae	35.09	WP_207292265.1
Lelliottia		
L. amnigena	91.39	WP_239608463.1
L. aquatilis	35.77	WP_103946518.1
L. jeotgali	35.77	ASV56541.1
L. nimipressuralis	91.06	WP_194512291.1
L. sp. AC1	32.41	WP_248860173.1
Mangrovibacter		
M. phragmitis	85.95	WP_064600681.1
M. plantisponsor	54.64	WP_110026257.1
M. sp. MFB070	32.64	WP_036114364.1
M. yixingensis	85.62	WP_226571755.1
Photobacterium		
P. alginatilyticum	35.23	WP_160652990.1
P. angustum	33.45	WP_005363905.1
P. aquae	35.46	WP_047879781.1
P. aquimaris	34.40	WP_060999368.1
P. atrarenae	36.40	WP_255391925.1
P. damselae	33.33	WP_065171315.1
P. frigidiphilum	32.75	WP_107245069.1
P. ganghwense	32.76	WP_217394300.1
P. halotolerans	34.39	WP_046220204.1
P. indicum	34.28	WP_107253231.1
P. jeanii	32.76	WP_068328270.1
P. leiognathi	33.57	WP_107235256.1
P. lipolyticum	33.10	WP_107282128.1

P. lucens	32.20	WP_161118504.1
P. lutimaris	32.65	WP_107346884.1
P. marinum	33.45	WP_007463377.1
P. phosphoreum	34.98	WP_107238999.1
P. profundum	32.40	WP_006229249.1
P. proteolyticum	32.29	WP_075765179.1
P. rosenbergii	36.97	WP_222552449.1
P. salinisoli	34.74	WP_120512884.1
P. sanguinicancri	30.82	WP_062690377.1
P. swingsii	31.25	WP_048899733.1
Phytobacter		
P. diazotrophicus	89.97	TCW43122.1
P. massiliensis	89.33	WP_044179493.1
P. palmae	91.33	SFE07833.1
P. sp. SCO41	34.72	WP_108700761.1
P. ursingii	90.33	VTP17027.1
Raoultella		
R. ornithinolytica	90.33	WP_132513533.1
R. planticola	89.67	WP_099001527.1
R. terrigena	89.33	MCF6690643.1
Salinivibrio		
S. costicola	31.99	WP_102505282.1
S. kushneri	31.99	WP_077653133.1
S. proteolyticus	33.00	WP_077674845.1
S. sharmensis	31.99	WP_077772380.1
S. siamensis	27.7	WP_077667813.1
S. socompensis	31.99	WP_025739776.1
Salmonella		
S. enterica	95.71	EEI0561811.1
	95.05	SUG22760.1
Vibrio		
V. alginolyticus	34.86	WP_224913632.1
V. azureus	34.62	AUI87663.1
V. cidicii	36.14	WP_061897803.1
V. coralliilyticus	36.59	WP_021455058.1
V. galatheae	35.69	WP_045955835.1
V. gigantis	36.01	WP_086714757.1
V. harveyi group	35.09	WP_194709782.1
V. natriegens	35.44	WP_255202947.1
V. neptunius	36.24	WP_206370971.1

V. parahaemolyticus	93.40	KKF68878.1
V. sp. Isolate30	37.94	WP_239842864.1
V. vulnificus	36.33	WP_238834538.1
Yersinia		
Y. aldovae	80.95	WP_004701445.1
Y. aleksiciae	81.97	WP_048617983.1
Y. alsatica	82.37	WP_145507836.1
Y. bercovieri	81.63	WP_145519672.1
Y. canariae	82.31	WP_145555162.1
Y. enterocolitica	82.31	WP_050159623.1
Y. entomophaga	37.02	OWF88311.1
Y. frederiksenii	81.97	WP_050318565.1
Y. hibernica	80.95	WP_129198693.1
Y. intermedia	82.31	WP_145546563.1
Y. kristensenii	80.95	WP_032820763.1
Y. massiliensis	81.97	WP_145495505.1
Y. mollaretii	82.31	WP_145573127.1
Y. nurmii	37.02	WP_049600163.1
Y. pekkanenii	80.95	WP_049612631.1
Y. pestis	82.52	WP_016671719.1
Y. pestis subsp. pestis	80.20	MBI0221253.1
Y. pseudotuberculosis	81.02	WP_050092824.1
Y. rochesterensis	81.29	WP_145589324.1
Y. rohdei	81.29	WP_054883122.1
Y. ruckeri	78.91	WP_004719344.1
Y. similis	81.36	WP_025381624.1
Y. thracica	81.29	WP_050116504.1
Y. vastinensis	81.63	WP_145580120.1
Y. wautersii	83.65	WP_033848408.1

Supplementary Table 5 Bacterial strains and plasmids used in this study[a]

Strain or plasmid	Phenotype and/or characteristic(s)	Source or reference
Strains		
Candida albicans SC5314	Wild-type strain of *Candida albicans*	Laboratory collection
Shigella sonnei CMCC51572	Wild-type strain of *Shigella sonnei*	Laboratory collection
Δ*ubiC*	*S. sonnei* with *ubiC* being deleted	This study
Δ*ubiC*(*ubiC*)	Mutant *ubiC* harboring the expression construct *pUC-ubiC*	This study
Δ*ubiC*(*aaeR*)	Mutant *ubiC* harboring the expression construct *pUC-aaeR*	This study
Δ*aaeR*	*S. sonnei* with *aaeR* being deleted	This study
Δ*aaeR*(*aaeR*)	Mutant *aaeR* harboring the expression construct *pUC-aaeR*	
Δ*ubiC*Δ*aaeR*	*S. sonnei* with *ubiC* and *aaeR* being deleted	This study
S. sonnei (P*ubiC-lux*)	*S. sonnei* harboring the reporter construct P*ubiC-lux*	This study
Δ*ubiC*(P*ubiC-lux*)	Mutant *ubiC* harboring the reporter construct P*ubiC-lux*	This study
Δ*aaeR*(P*ubiC-lux*)	Mutant *aaeR* harboring the reporter construct P*ubiC-lux*	This study
S. sonnei (P*trpE-lux*)	*S. sonnei* harboring the reporter construct P*trpE-lux*	This study
Δ*ubiC* (P*trpE-lux*)	Mutant *ubiC* harboring the reporter construct P*trpE-lux*	This study
Δ*aaeR* (P*trpE-lux*)	Mutant *aaeR* harboring the reporter construct P*trpE-lux*	This study
S. sonnei (P*aaeR-lux*)	*S. sonnei* harboring the reporter construct P*aaeR-lux*	This study
Δ*ubiC* (P*aaeR-lux*)	Mutant *ubiC* harboring the reporter construct P*aaeR-lux*	This study
E.coil DH5α	*supE44 lacU169(80lacZ M15) hsdR17 recA1 endA1 gyrA96 thi-1 relA1 pir*	Laboratory collection
E.coil BL21(DE3)	*F-ompT hsdS (rB-mB-) dcm+ Tetr gal (DE3) endA*	Laboratory collection
Plasmids		
pUC18	Broad-host-Range cloning vector, Amp^r	Laboratory collection
pET28(a)+	Expression vector, Kan^r	Laboratory

		collection
pKD3	Template for amplifying the *cat* gene	Laboratory collection
pKD46	λ Red recombinase expression, Amp^{r}	Laboratory collection
pCP20	FLP recombinase expression, Amp^{r}	Laboratory collection
pMS402	Expression reporter plasmid carrying the promoterless *LuxCDABE*	Laboratory collection
pET-UbiC	pET28 containing *ubiC*	This study
pET-AaeR	pET28 containing *aaeR*	This study
P*trpE-lux*	pMS402 containing the promoter of *trpE*	This study
P*aaeR-lux*	pMS402 containing the promoter of *aaeR*	This study
P*ubiC-lux*	pMS402 containing the promoter of *ubiC*	This study

[a]Kan^{r}, resistance to kanamycin; Amp^{r}, resistance to ampicillin;

Supplementary Table 6 PCR primers used in this study[a]

Primer	Sequence (5'-3')
For deletion	
ubiC-KO-F	CGATACAATGCCTTTACGTTATGTAACGGAGAGTTCGGCGTGTAGGCTGGAGCTGCTTC
ubiC-KO-R	CTTATTCTGCGTCAGACTCCACTCCATATTTTTTTCCTCATGGGAATTAGCCATGGTCC
aaeR-KO-F	ACTTTTAAGTCAGAGTGAATAATGGAACGACTAAAACGCGTGTAGGCTGGAGCTGCTTC
aaeR-KO-R	GTTGGCACAGGTTTGTAGAACGTAACAGTACAATATGAAATGGGAATTAGCCATGGTCC
For *in trans* expression	
ubiC-pUC-F	CGGAATTCATGCGATTGTTGCGTTTTT
ubiC-pUC-R	CGGGATCCTTAGTACAACGGTGACGCC
aaeR-pUC-F	CGGAATTCATGTCGGTGTTTGCCAAAGT
aaeR-pUC-R	CGGGATCCTTACTTCTCTTTCCCGCGC
For reporter	
P*ubiC*-lux-F	CCGCTCGAGGCCTTACATGATATGTTGAATCC
P*ubiC*-lux-R	CGGGATCCTAACGCGGGGTGTGACAT
P*trpE*-lux-F	CCGCTCGAGAAGGCGCACTCCCGTTCTG
P*trpE*-lux-R	CGGGATCCTGCTTTCATTGTCGATACCCT
P*aaeR*-lux-F	CCGCTCGAGGGAAAGCCGGGCGAGAGC
P*aaeR*-lux-R	CGGGATCCAAATTCAACTACTTTGGCAAAC
For EMSA	
ubiD-EMSA-F	AATGACTCTTATCCGTTTAATCG
ubiD-EMSA-R	ATATTTCATGGCGTCCATTG
ubiC-EMSA-F	GCCTTACATGATATGTTGAATCC
ubiC-EMSA-R	TAACGCGGGGTGTGACAT
For recombinant protein expression	
AaeR-P28-F	CGCGATATCGTCGACGGATCCATGTCGGTGTTTGCCAAAGTA
AaeR-P28-R	GCCCTTGCTCACCATGAATTCCTTCTCTTTCCCGCGCCC
UbiC-P28-F	CGGAATTCATGCGATTGTTGCGTTTTT
UbiC-P28-R	CGGGATCCTTAGTACAACGGTGACGCC
For deletion confirmation	
Out-*ubiC*-F	ATCATGGGCGGTGATGC
Out-*ubiC*-R	CCGTACGCATTAAGCGAT
In-*ubiC*-F	ATGTCACACCCCGCGTTAAC
In-*ubiC*-R	TTAGTACAACGGTGACGCCGG
Out-*aaeR*-F	CACTTTTAAGTCAGAGTGAATA
Out-*aaeR*-R	GGCACAGGTTTGTAGAACGT
In-*aaeR*-F	ATGTCGGTGTTTGCCAAAGTAGT

In-*aaeR*-R	CTTCTCTTTCCCGCGCCC
For RT-qPCR analysis	
S. sonnei	
trpE-F	CAACGCTGCTGCTGGAAT
trpE-R	GTGTCACCTAATGCTGTAATGC
trpD-F	GTGACGGCAGCAACAGTATC
trpD-R	CAGCAGATCGGACGAACCA
narK-F	TTGCCGTGCAGGATACCT
narK-F	ATGTTTCCCAGACCACCA
rplP-F	CGGATGTTAGCTTCGGCA
rplP-R	CAGCGGCTTTTCAGTGATC
ubiD-F	TGGATCCGCATCTGGAAAT
ubiD-R	TACCAACTTCACGCAGCG
rplV-F	CTATCGCTAAACATCGCCA
rplV-R	CAGCGTTAGCAATGGCAGA
ssuA-F	CCGCAAATGTTGGAAGCG
ssuA-R	TTCTGCCACCAGAATCACT
degP-F	AACCGTTAATACGCCGCG
degP-R	AATGATGACGCCGGAACC
abgA-F	CACTATGCAGAGTCTGGC
abgA-R	TGTTGACGAGCGCGCTC
ccmE-F	TGGCGCTGACTATCGGTC
ccmE-R	ACACTACCCAGCATCACCA
osmB-F	GGCAATGTCTCTGAGTGC
osmB-R	CCTAATGTACCCAACGTACT
napB-F	CTGTGTCAATGGACGGCG
napB-R	TTGGCATCCGAATGGCCC
nirD-F	AATCGATGACATCCTGCCT
nirD-R	GATAGCACGCTGGACTCG
abgB-F	TCAGCGGAGCATCTGGC
abgB-R	CCAGCAGGGCGATAACC
pyrL-F	TGGTTCAGTGTGTTCGACA
pyrL-R	ATCTCCTGACGCCTGGG
rpsQ-F	CGTACTCTGCAAGGTCGC
rpsQ-R	CTCGTCATGTACGTGCAG
nac-F	ATCCATCACCATGCCCTT
nac-R	AATCACCGCCATATCGAG
rpsS-F	GCCACGTTCTCTCAAGAAA
rpsS-R	GCGATGGTCAAACCGATCA
nikC-F	TGTCGCTCGGTTCGGTAA
nikC-R	CGGGAAGGTCATAAACATA
aaeR-F	GGATGCTTCATGAAGTGCA
aaeR-R	TTTGCTGTCAGCCCGGC
rpmI-F	GGTGCTGCTAAGCGCTT

rpmI-R	TTTCGGACGCAGGTGACG
hisG-F	CGTATCGCCACCTCTTATCCT
hisG-R	TCAACAGAACCGTTCAGTAAGC
C. albicans	
Gsp1-F	TGAAGTCCATCCATTAGGAT
Gsp1-R	ATCTCTATGCCAGTTTGGAA
Hwp1-F	TGGTGCTATTACTATTCCGG
Hwp1-R	CAATAATAGCAGCACCGAAG
Efg1-F	TATGCCCCAGCAAACAACTG
Efg1-R	TTGTTGTCCTGCTGTCTGTC
Als3-F	CTAATGCTGCTACGTATAATT
Als3-R	CCTGAAATTGACATGTAGCA
Cst20-F	TTCTGACTTCAAAGACATCAT
Cst20-R	AATGTATATTTCTGGTGGTG
Cdc35-F	TTCATCAGGGGTTATTTCAC
Cdc35-R	CTCTATCAACCCGCCATTTC
Tec1-F	AGGTTCCCTGGTTTAAGTG
Tec1-R	ACTGGTATGTGTGGGTGAT
Cph1-F	ATGCAACACTATTTATACCTC
Cph1-R	CGGATATTGTTGATGATGATA
Ece1-F	GCTGGTATCATTGCTGATAT
Ece1-R	TTCGATGGATTGTTGAACAC
Pde2-F	ACCACCACCACTACTACTAC
Pde2-R	AAAATGAGTTGTTCCTGTCC
Hst7-F	ACTCCAACATCCAATATAACA
Hst7-R	TTGATTGACGTTCAATGAAGA
Cek1-F	TTAGAAATTGTTGGAGAAGGAGCAT
Cek1-R	GCAACTTTTTGTTGTGATGGTTTATG
Rim101-F	TGGCAATGGCTCATACAC
Rim101-R	ACACCGCCAAACTCTAAT
Hsp90-F	GCTTTAAGTGCTGGTGCT
Hsp90-R	CGTATTGTTCGTCGTCAT
Ras1-F	ATCCGCTTTAACCATTCA
Ras1-R	TCTTGTCCAGCAGTATCT
Als1-F	ACAACAGGCACCTCAGCA
Als1-R	GAACCAGAGCCATCGTAT

[a]Restriction enzyme sites are underlined. EMSA, electrophoretic mobility shift assay; F, forward; R, reverse; RT, reverse transcription.
